# Real-time evaluation of the independent analgesic efficacy of dexmedetomidine

**DOI:** 10.1186/s12871-023-02022-2

**Published:** 2023-03-04

**Authors:** XiaoHua Wang, SiYuan Zhang, Chunxiu Wang, Yi Huang, Hao Wu, Guoguang Zhao, TianLong Wang

**Affiliations:** 1 Department of Anesthesiology, Xuanwu Hospital, Capital Medical University, Beijing, 100053 China; 2 National Clinical Research Center for Geriatric Disorders, Beijing, China; 3 Daxing Hospital Affiliated to Capital Medical, Daxing District, Xingfeng Street, Beijing, 100026 China; 4 Department of Evidence-Based Medicine, Xuanwu Hospital, Capital Medical University, Beijing, China; 5 Department of Neurosurgery, Xuanwu Hospital, Capital Medical University, Beijing, 100053 China

**Keywords:** Dexmedetomidine, Analgesics, Nociception index, IOC2, qNOX

## Abstract

**Background:**

Dexmedetomidine has analgesic properties, but the intraoperative analgesic effect of dexmedetomidine is often masked by the effects of other general anaesthetics. Therefore, the degree to which it reduces intraoperative pain intensity remains unclear. The objective of this double-blind, randomised controlled trial was to evaluate the independent intraoperative analgesic efficacy of dexmedetomidine in real-time.

**Methods:**

This single-centre study enrolled 181 patients who were hospitalised for below-knee orthopaedic surgeries between 19 January 2021 to 3 August 2021 were eligible for this is single-centre study. Peripheral neural block was performed on patients scheduled for below-knee orthopaedic surgeries. Patients were randomly assigned to the dexmedetomidine or midazolam group and were intravenously administered with 1.5 µg kg^−1^ h^−1^ dexmedetomidine or 50 µg kg^−1^ h^−1^ midazolam, respectively. The analgesic efficacy was evaluated using the real-time non-invasive nociception monitoring. The primary endpoint was the attainment rate of the nociception index target. The secondary endpoints included the occurrence of intraoperative hypoxemia, haemodynamic parameters, the consciousness index, electromyography and patient outcomes.

**Results:**

On Kaplan–Meier survival analysis, the defined nociception index target was attained in 95.45% and 40.91% of patients receiving dexmedetomidine and midazolam, respectively. Log-rank analysis revealed that the dexmedetomidine group attained the nociception index target significantly faster and the median attainment time of the nociception index target in the dexmedetomidine group was 15 min. Dexmedetomidine group was associated with a significantly lower incidence of hypoxemia. There was no significant difference in blood pressure between the dexmedetomidine and midazolam groups. Further, the dexmedetomidine group had a lower maximum visual analogue scale score and lower analgesic consumption postoperatively.

**Conclusions:**

Dexmedetomidine has independent analgesia and systemically administered as an adjuvant agent has better analgesic efficacy than midazolam without severe side effects.

**Trial registration:**

clinicaltrial.gov Registry Identifier: NCT-04675372.Registered on 19/12 /2020.

## Introduction

Dexmedetomidine (DEX), a highly selective a2-adrenoreceptor agonist, is a relatively new adjuvant drug with sympatholytic, anxiolytic and analgesic properties [1]. As a key adjuvant of multimodal analgesia, DEX can significantly enhance the analgesic effect of general anaesthetics even at a low dose and can effectively inhibit opioid pain hypersensitivity and analgesic tolerance [2]. Furthermore, DEX has the beneficial property of an analgesia-sparing effect [3]. Moreover, it provides good intraoperative analgesia, reduces postoperative pain and opioid requirements, helps attain stable haemodynamic conditions and has minimal side effects [4]. It is widely used in clinical practice for its analgesic effect [5]. However, the intraoperative analgesic effect of DEX is often masked by the effects of other general anaesthetics. Therefore, the degree to which DEX reduces intraoperative pain intensity remains unclear, and its adverse effects during the maintenance period have not been fully elucidated.

Patients hospitalised for fractures have a high risk of pain and anxiety [6]. Pain can accentuate the body’s stress response and adversely affect endocrine and immune functions [7, 8]. Peripheral neural block (PNB) is the preferred technique and standard of anaesthesia care for below-knee orthopaedic surgeries. The systemically administered DEX as an adjuvant analgesic during PNB can prevent disturbances from other general anaesthetics, act on distinct pharmacologic sites and exhibit its independent anti-nociceptive effect.

Another challenge in analgesia is objectively monitoring the analgesia level in real-time to evaluate pain in an unconscious patient. Most trials have been limited by the use of visual analogue scales to evaluate the level of analgesia; however, this is subjective and cannot be used in deeply sedated patients. Our recent studies have shown that the nociception index (IOC2, namely qNOX), a new real-time monitoring index, responds to external noxious stimuli and thus allows us to objectively evaluate analgesic effects and pain intensity in patients under general anaesthesia [9–12].

This study aimed to quantitatively evaluate the analgesic effects of DEX using IOC2 by determining the rate and time required to attain the target IOC2 (i.e. mean optimal analgesia) in patients who underwent below-knee orthopaedic surgery. Further, the safety and postoperative efficacy of DEX were assessed.

## Materials and methods

### Ethics

This trial was approved by the independent Institutional Ethics Committee of Xuanwu Hospital (Chairperson Prof. Yanghai Cui, identifier: IRB-XWAD-202008–12) on 12 /08/2020. The trial was also registered in the ClinicalTrial.gov registry (identifier: NCT04675372)(Sponsors: China International Neuroscience Institution) on 19/12 /2020. This randomised, double-blind, parallel-group clinical trial was designed, implemented, executed and overseen by the study sponsor and steering committee. Written informed consent was obtained from all study participants before enrolment. The study was conducted in accordance with the good clinical practice guidelines indicated in the Declaration of Helsinki and relevant regulatory requirements [13].

### Study participants and randomisation

In this prospective study, patients presented to our institution with below-knee fractures and were hospitalised for below-knee orthopaedic surgeries. Using a computer-generated randomisation schedule, the patients were randomly assigned (1:1) to the DEX or midazolam (MID) group according to the allocation sequence.

The inclusion criteria of the study were as follows: (1) patients undergoing below-knee orthopaedic surgeries and internal fixation under PNB anaesthesia, (2) aged 18–80 years, (3) classified using ASA (American Society of Anesthesiologists) grade I–IV, (4) having a body mass index (BMI) of 18.5–35 kg m^−2^and (5) underwent surgeries that lasted for < 3 h.

The study excluded patients (1) with a history of chronic use of alcohol, opioids or other sedative drugs; (2) with a history of allergy to any medications used in this study; (3) with severe arrhythmia; (4) who underwent PNB that was ineffective; (5) with preoperative systolic blood pressure (SBP) of < 85 mmHg; (6) with a preoperative heart rate (HR) of < 45 bpm; (7) with Alzheimer’s disease; (8) with epilepsy; (9) diagnosed with mental illness or other autonomic nervous system disorders that may affect electroencephalogram (EEG) findings and (10) underwent surgeries that lasted for > 3 h.

### Neuromonitoring methods

The level of analgesia was recorded continuously using the proprietary index IOC2 (Index of consciousness 2,namely qNOX) (Angel-6000D Multi-parameter Anesthesia Monitor, Shenzhen Weihaokang Medical Technology Co., Ltd, Guangdong, China). IOC2was used in this study to estimate the responses of patients to noxious stimuli and analgesic levels [9–12]. IOC2 is also known as qNOX (CONOX monitor, Fresenious Kabi/Quantium Medical) in the European market. IOC2 values of < 90, which were the target of this study, indicate adequate analgesic effect and analgesic level for regional surgery. IOC2 values of ≥ 90 indicate inadequate use of analgesics, whereas IOC2 values of < 20 suggests excessive use of analgesics. IOC2 and the consciousness index (IOC1) are dimensionless indices ranging continuously from 99 to 0. IOC1, which is highly correlated with other hypnotic indices such as the bispectral index [14], is a combination of different frequency bands of EEG fed into a quadratic model, which generates an output fitted to hypnotic-related variables. The IOC1 values close to 99 represent the ‘awake’ state, whereas those close to 0 denote an isoelectric EEG. Concurrently, we recorded the degree of muscle relaxation according to the electromyography (EMG) response and the corresponding signal quality index, which reflected the stability of the monitoring process.

### Anaesthetic procedures

Upon enrolment, the patients arriving at the operation theatre without premedication were given 8 ml kg^−1^ Ringer’s solution via an intraoperative maintenance infusion of 4 ml kg^−1^ h^−1^. Standard physical monitoring was performed using an automated non-invasive blood pressure (BP) monitor, 5-lead ECG and pulse oximetry. Systolic BP (SBP), diastolic BP (DBP), mean arterial pressure (MAP) and HR were recorded at intervals of 5 min during the entire operation. To objectively record the baseline parameters, baseline measurements were defined using the average of three readings obtained at an interval of 5 min before induction in the supine position on the operation bed.

PNB (femoral and sciatic nerve blocks) was performed under ultrasound guidance combined with a nerve stimulator (MultiStim SENSOR, PAJUNK, Geisingen, Germany). If electrical stimulation of ≤ 0.5 mA elicited a visible motor response in the quadriceps femoris for femoral nerve or in the gastrocnemius for sciatic nerve, approximately 20 ml of ropivacaine hydrochloride (3.5 mg ml^−1^) (Naropin, AstraZeneca AB, Sodertalje, Sweden) was injected. The block was considered satisfactory after confirming the presence of complete motor and sensory blocks. The presence of a motor block was assessed using the modified Bromage scale for the lower limb (0: normal motor function; 1: ability to only move the toes; and 2: inability to move the knee, ankle and toes), with a Bromage score of 2 indicating a complete block. The presence of a sensory block was assessed via the pin-prick method using a 26G hypodermic needle along the midline of the lower limb [15]. A successful sensory block was defined as a complete lack of pain sensation at the surgical field level. Patients who successfully achieved a complete block were randomly administered with 1.5 µg kg^−1^ h^−1^ DEX [16] (H20090248, Jiangsu Hengrui Pharmaceuticals Co., Ltd, Lianyungang, Jiangsu, China) or 50 µg kg^−1^ h^−1^MID (H10980025, Jiangsu Nhwa Pharmaceutical Co., Ltd, Xuzhou, China) [17]. The drug dosage was calculated according to the lean body weight (LBM), and the drugs were continuously administered during the procedure until wound irrigation. The parameters were recorded even after the operation was completed.

During inhalation of air, side effects such as hypotension (SBP < 90 mmHg or DBP < 60 mmHg), bradycardia (HR < 55 bpm) and hypoxemia (SpO_2_ level < 93%) were observed and noted. An SpO_2_ level of < 93% was treated with 2–4 l min^−1^ oxygen administration. Hypotension was treated with 6 mg of intravenous ephedrine administration. Further, sinus bradycardia was treated with 0.5 mg of intravenous atropine administration. These side effects were reported by the anaesthesiologist who was blinded to the study protocol.

### Study endpoints

The primary endpoint of the study was the attainment rate of the IOC2 target during operation. The secondary endpoints were associated with the composite rate of major adverse effects, including the incidence of hypoxemia (i.e. respiratory depression), haemodynamic changes (i.e. hypotension and bradycardia), EMG activity and IOC1. The patients received instructions for using a 10 cm visual analogue scale (VAS) to assess pain [VAS 0 (no pain) to VAS 10 (the worst possible pain)] once daily postoperatively. The maximum VAS (VAS_MAX_) score indicates the maximal postoperative VAS score of each patient after operation during hospitalisation. Analgesia consumption was used to describe oxycodone accumulation (oral administration of oxycodone 5 mg for each complaint of pain) postoperatively. The functional recovery and hospital stay were also assessed.

### Blinding and quality control

**Fig. 1:**
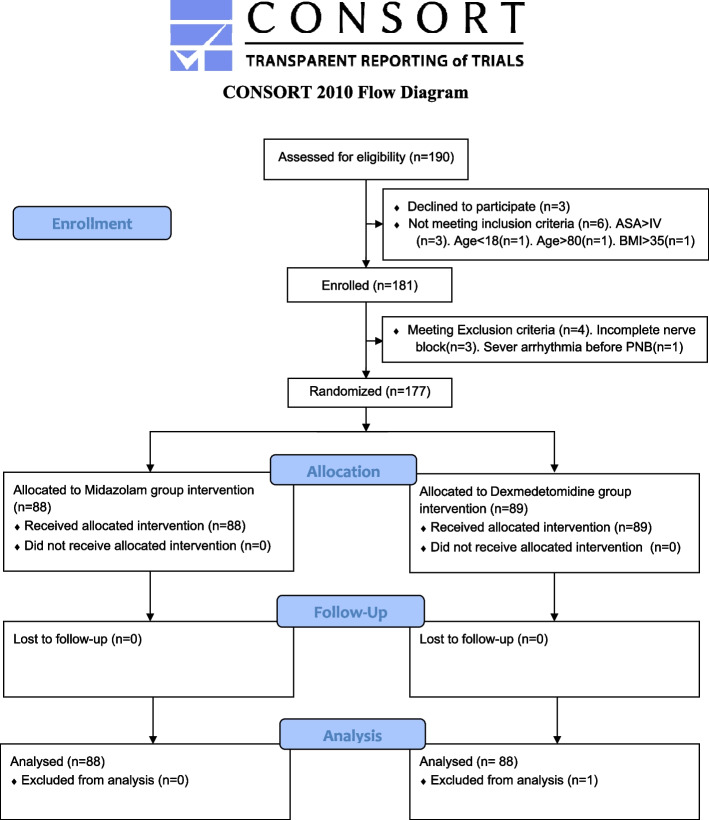
Patient selection flowchart

Using sealed opaque envelopes, patients were randomly assigned to the DEX or MID group according to a computer-generated randomisation schedule (Fig. 1). One research assistant received the sealed envelopes for each patient. To maintain masking of the distribution, DEX and MID were diluted to 50 ml using a 50-ml syringe with an infusion speed of 50 ml h^−1^. The concentration was calculated according to the patient’s LBM, and the solutions were prepared by the research assistant. PNB was performed by the same anaesthesiologist who was blinded to the procedure. The block effects were assessed by another anaesthesiologist who was also blinded to the treatment group. All measurements and recordings during the operation were noted by an observer who was blinded to the treatment drug administered to the patient. Anaesthesia implementer, data recorders, patients and result analysts were unaware of the groupings. To monitor safety, adverse events were recorded during all blinded drug administrations. The principal investigators of the trial accept full responsibility for the accuracy and completeness of the reported data analyses and interpretations that were performed independently. Another independent data and safety monitoring board had access to the unblinded data periodically that reviewed the safety results and was responsible for total quality control.

### Statistical analyses

In the pilot study, the sample size was calculated based on the objective of assessing differences of the attainment rate of the IOC2 target because it was the primary outcome. A power analysis revealed that 69 individuals per treatment group would provide at least 90% power with a significance level of 0.05 (two-tailed) to detect a 50% relative reduction in the attainment rate of the IOC2 target. Considering a 20% loss, at least 83 patients were required in each group.

Statistical analyses were performed using the R v3.0.1 software (http://www.Rproject.org). Kaplan–Meier (K–M) survival analysis was conducted to compare the efficacy of different interventions in attaining the target IOC2 as well as in analysing the time required to attain the target IOC2. Log-rank test was used to compare different K–M curves. The median cumulative and mean rank of hypoxemia and other skewed data were compared using the Mann–Whitney U test (independent samples). Normally distributed data are expressed as mean [standard deviation (SD)]. Group comparisons for normally distributed variables were performed using the *t*-test. Repeated analysis of variance was performed, followed by post-hoc pairwise comparisons for mean differences. All reported *P*-values were two-sided, and statistical significance was set at *P* < 0.05.

## Results

### Patient population

The dataset included patients from 19 January 2021 to 3 August 2021. A total of 190 patients in clinic were hospitalised and scheduled for elective surgery for below-knee fractures. Three cases were ineligible due to refusal to participate. Further, we excluded six patients with the following characteristics: ASA > IV (one case of multiple organ failure and two cases of severe brain trauma after injury) (*n* = 3), age > 85 years (*n* = 1), age < 18 years (*n* = 1) and BMI > 35 kg m^−2^ (*n* = 1). Finally, a total of 181 patients were enrolled in the study. We further excluded four patients because of incomplete nerve block (*n* = 3) and severe arrhythmia on day of surgery (*n* = 1). A total of 177 patients were randomly assigned to 2 groups (89 and 88 patients in the DEX and MID groups, respectively). Due to missing data on one patient, the primary endpoint was available for 88 patients in the DEX group and 88 patients in the MID group. Figure 1 illustrates the patient selection flowchart. All patients were discharged safely after the operation.

### Patient demographics and baseline characteristics

Among the patients administered with DEX or MID, no significant differences were observed in demographics (i.e. age, sex, weight, height, BMI and ASA grade), baseline characteristics (i.e. SBP, DBP, MAP and HR), surgical distribution, volume of liquid infusion, blood loss, operation time and anaesthesia time (Table 1). None of the patients were excluded from the study due to serious complications.

**Table 1:** Patient demographics and baseline characteristics

Variation ^a^	DEX group	MID group	t/X^2^	*P-*value
Age, years	55.55 (14.98)	55.77 (12.92)	1.332	0.212
Height, cm	165.56 (9.45)	166.50 (7.86)	1.107	0.654
Weight, kg	69.82 (11.10)	68.37 (11.81)	1.509	0.073
BMI, kg/m^2^	25.41 (2.94)	24.57 (3.31)	1.400	0.139
Anaesthesia time, min	132.78 (42.74)	139.11 (50.47)	1.661	0.086
Operation time, min	82.28 (36.38)	86.56 (44.69)	1.971	0.093
Bleeding, ml	39.73 (50.43)	29.55 (34.45)	2.321	0.079
Intraoperative infusion, ml	534.66 (230.36)	535.25 (221.81)	1.225	0.393
Baseline SBP, mmHg	143.92 (29.11)	136.75 (30.30)	1.438	0.364
Baseline DBP, mmHg	83.50 (11.21)	79.17 (11.02)	1.035	0.950
Baseline MAP, mmHg	99.94 (15.54)	95.33 (13.18)	1.390	0.412
Baseline HR, beats/min	75.56 (14.89)	73.58 (12.56)	1.405	0.396
Variation ^b^				
ASA score I–II (%)	65 (73.86)	74 (84.09)	2.772	0.096
ASA score III–IV (%)	23 (26.14)	14 (15.91)		
Female sex (%)	53 (60.23)	45 (51.14)	1.474	0.225
Male sex (%)	35 (39.77)	43 (48.86)		

^a^ Data are expressed as mean (SD)

^b^ Data are expressed as number (percentage)

*DEX* Dexmedetomidine, *MID* Midazolam, *SBP* Systolic blood pressure, *DBP* Diastolic blood pressure, *MAP* Mean arterial pressure, *HR* Heart rate

### Primary endpoints

**Fig. 2:**
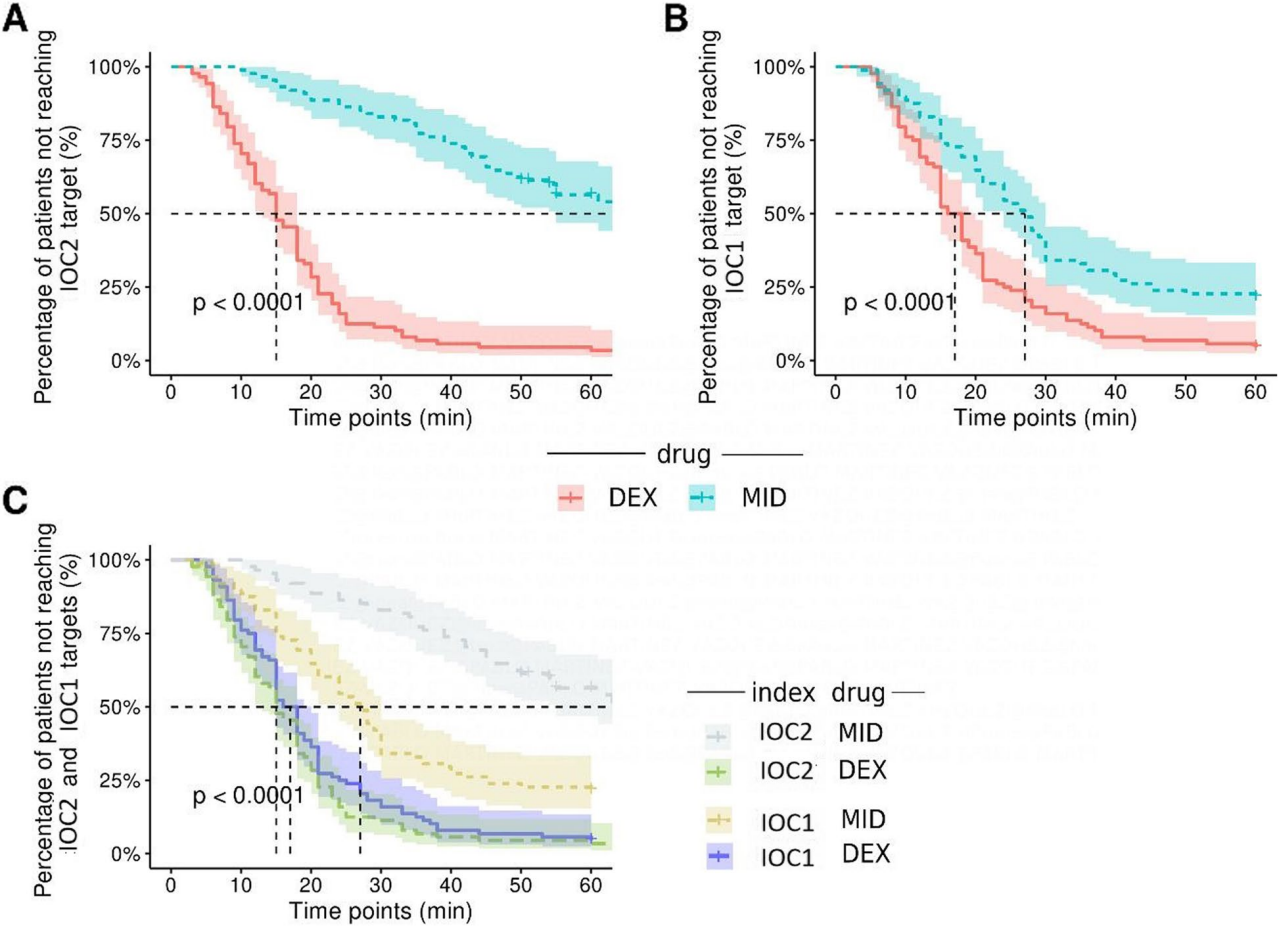
**A** Kaplan–Meier curves analysing the onset of the target non-invasive nociception index (IOC2) after continuous infusion of dexmedetomidine (DEX) or midazolam (MID). The graph shows a significantly faster onset of the target IOC2 in the DEX group than in the MID group (log-rank test). The DEX group had a higher probability of attaining the target IOC2 than the MID group. The y-axis denotes the percentage of patients that have not attained that IOC2 target, as detected by a log-rank percentage, whereas the x-axis denotes the time from drug administration. The time to reach the consciousness index (IOC1) target decreased. **B** Kaplan–Meier curves analysing the onset of the target IOC1. The y-axis denotes the percentage of patients that have not reached the IOC1. The x-axis denotes the time from the administration of the drug. **C** Kaplan–Meier analysis reflecting the different indices for the DEX and MID groups attaining 50% at different time points

The primary endpoint of IOC2 was assessed in terms of the rate to attain the target value. The IOC2 target was attained in 84 of 88 patients (95.45%) in the DEX group and 36 of 88 patients (40.91%) in the MID group. The K–M survival curve analysis revealed significant differences in the survival distributions between the DEX and MID groups (Fig. 2) (log-rank test, X^2^ = 114.0, df = 1, *P* < 0.001). The median attainment time of the IOC2 target was 15 min in the DEX group [Hazard ratio of MID/DEX: 0.174, 95% confidence interval [CI]: 0.117–0.258; HR of DEX/MID: 5.744, 95% CI: 3.871–8.524; *P* < 0.0001) (Fig. 2).

### Secondary endpoints

#### Hypoxemia occurrence

**Fig. 3:**
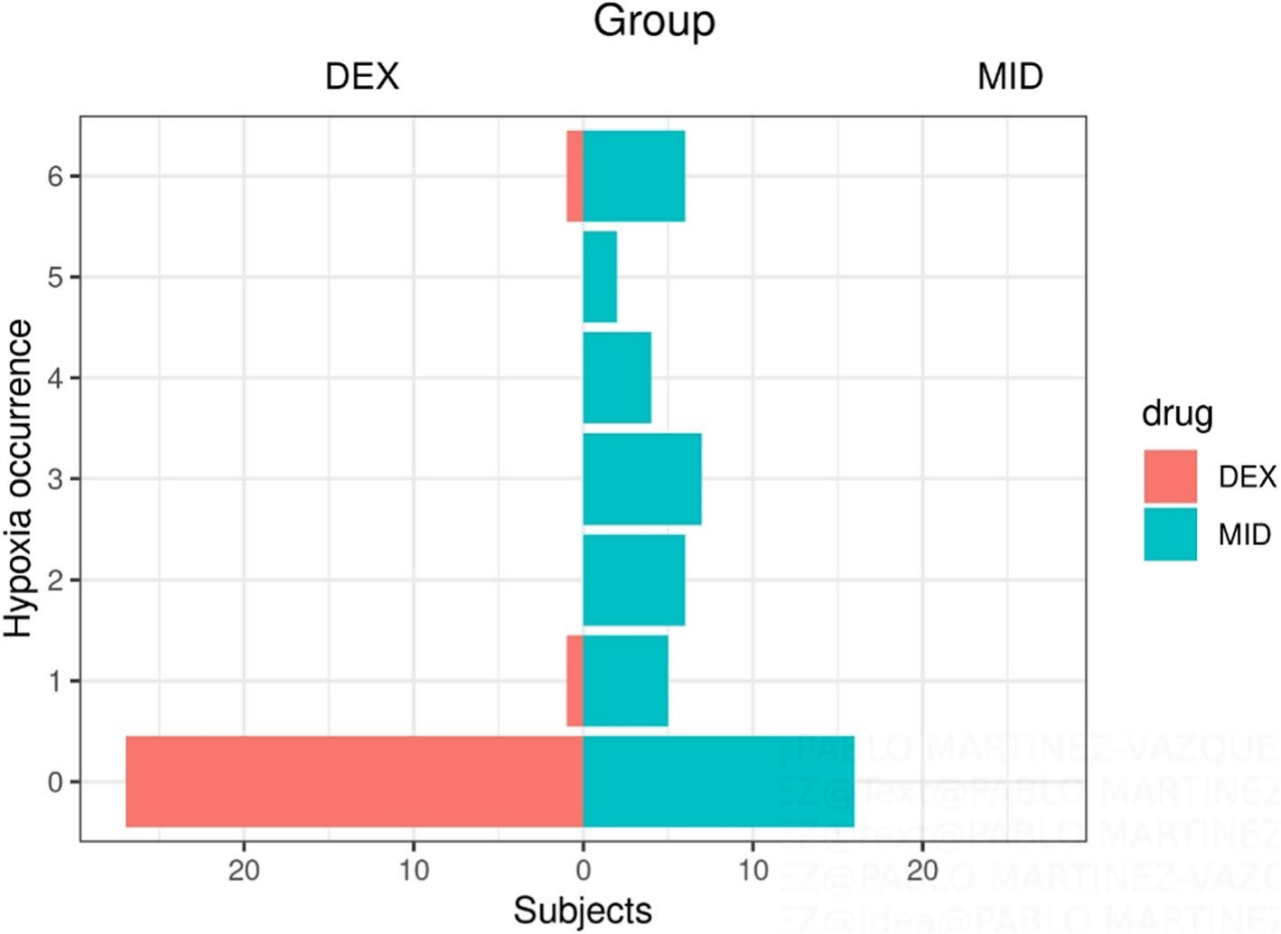
The population pyramid for the total frequency of hypoxemia in the dexmedetomidine (DEX) and midazolam (MID) groups. The distribution of hypoxemia in the DEX and MID groups was different as assessed by visual inspection of the population pyramids. The Mann–Whitney U test reflects the occurrence of hypoxemia

The DEX group had a significantly lower total incidence of hypoxemia than the MID group (5.68% vs 50%, *P* = 0.01). The summarised frequency of hypoxemia is shown in Fig. 3. The duration of hypoxemia was also significantly different between the DEX and MID groups (4 vs. 9.52 min). In the Mann–Whitney U test, the mean rank of hypoxemia was 69.98 for the DEX group and 107.02 for the MID group (Z =  − 6.106, *P* < 0.001).

### Haemodynamic evolution

**Fig. 4:**
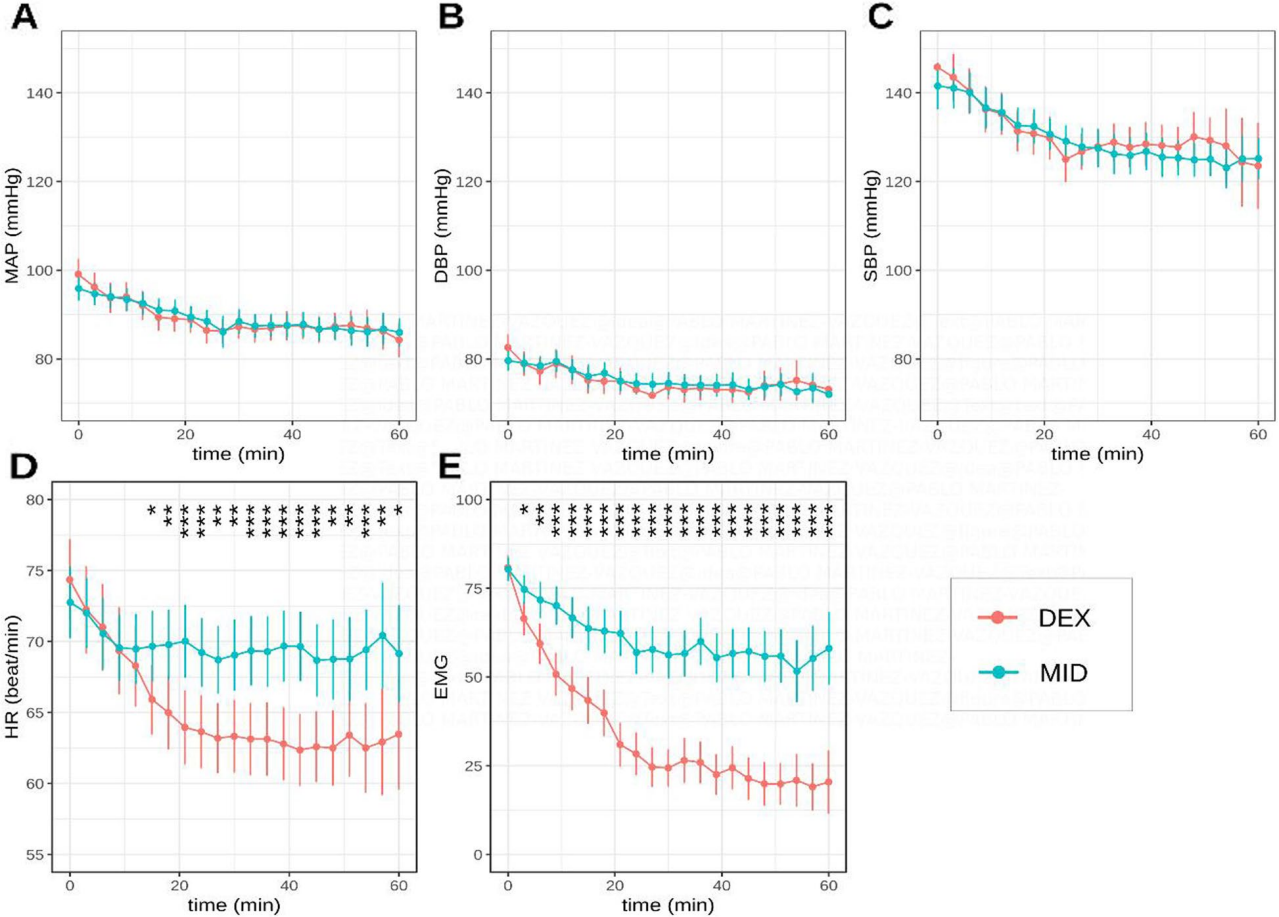
Mean arterial pressure (MAP), diastolic blood pressure (DBP), systolic blood pressure (SBP), heart rate (HR) and electromyography (EMG) measurements at different time points from induction. **A** MAP fluctuation, **B** DBP fluctuation, **C** SBP fluctuation, **D** HR fluctuation and (**E**) EMG fluctuation. * represent a statistical difference between the two groups. ***P < 0.001, **P < 0.01, *P < 0.05

Haemodynamic parameters were relatively stable during the procedure in both groups. During the surgical operation, SBP, DBP and MAP followed a descending trend; however, no significant differences were found between the two groups (Fig. 4A-C). The mean HR values decreased throughout the operation, with significantly lower values in the DEX group (Fig. 4D). But severe hypotension (MAP < 65 mmHg) and severe bradycardia (HR < 45 bpm) were not observed in any patient during the operation.

### Muscle relaxation evolution according to facial electromyography

The changes in EMG values, which reflect the degree of muscle relaxation, are shown in Fig. 4E. The DEX group have lower EMG values than the MID group at all time points except the first time point.

### IOC1 values

The median attainment time of the IOC1 target was 17 and 28 min in the DEX and MID groups, respectively (Fig. 2B). On K–M analysis, the IOC1 target was attained in 84 of 88 patients in the DEX group and 69 of 88 patients in the MID group (X^2^ = 19.81, *P* < 0.0001; HR of DEX/MID: 0.6296, 95% CI: 0.4579–0.8657; HR of MID/DEX: 1.588, 95% CI: 1.155–2.184) (Fig. 2C).

### Subgroup analysis

**Fig. 5:**
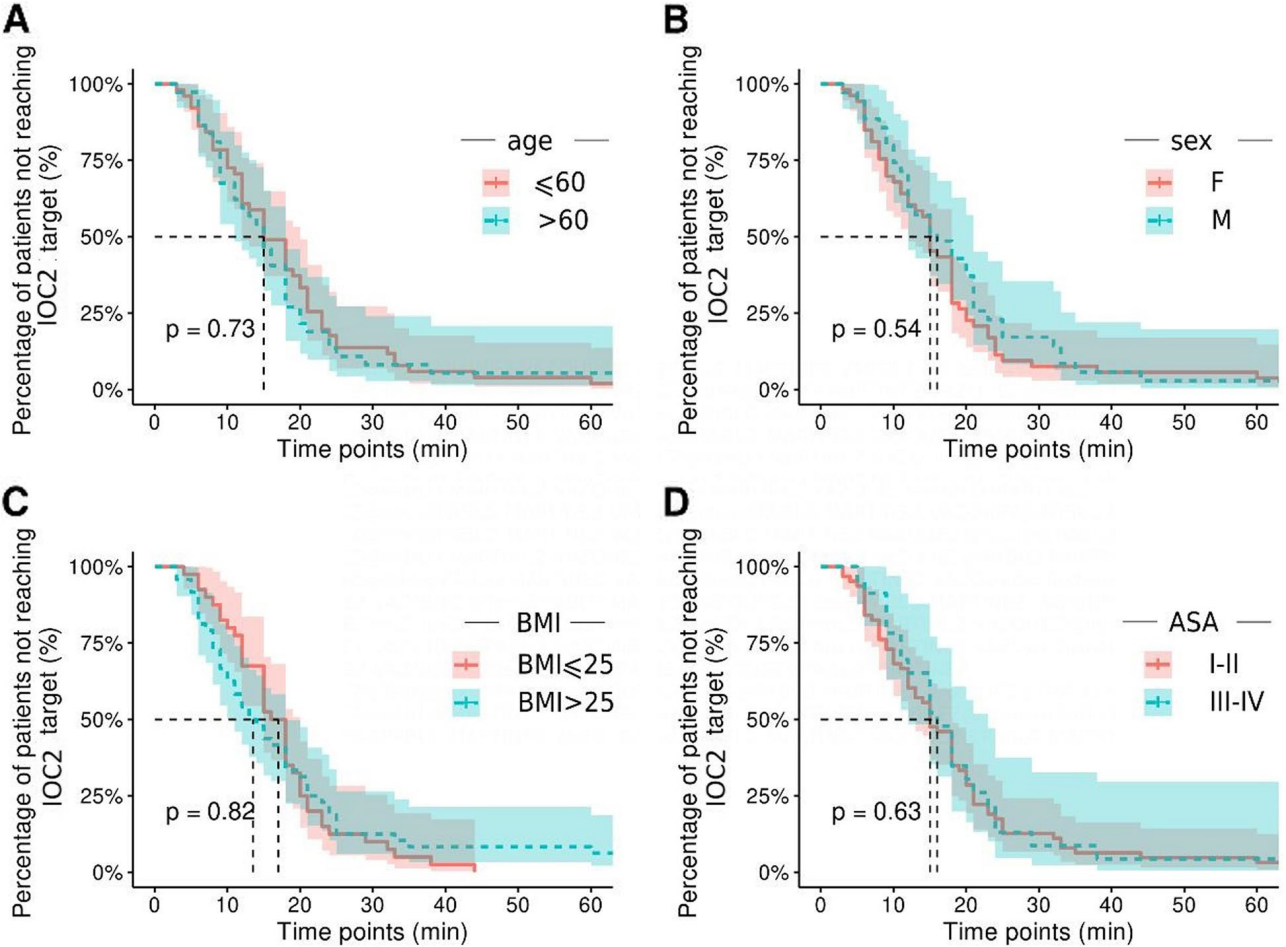
Kaplan–Meier curves within subgroups in the dexmedetomidine (DEX) group according to (**A**) age, (**B**) sex, (**C**) BMI, and (**D**) ASA stage

Subgroup analysis was performed to investigate the potential factors that affected the IOC2 target attainment in the DEX group. Among patients who attained the IOC2 target in the DEX group, we analysed their age, sex, BMI and ASA score. There was no significant difference in the attainment of the IOC2 target value during subgroup analyses for age: age ≤ 60 vs age > 60 years (*P* = 0.73) (Fig. 5A); sex: female vs male (*P* = 0.54) (Fig. 5B); BMI: BMI ≤ 25 vs BMI > 25 kg m^−2^ (*P* = 0.82) (Fig. 5C) and ASA: patients with ASA grade III–IV compared with patients with ASA grade I–II in the DEX group (*P* = 0.63) (Fig. 5D).

### Outcomes in patients treated with DEX versus MID

The DEX group had a significantly lower postoperative VAS_MAX_ score than the MID group [mean (SD): 0.28 (0.86) vs 2.26 (2.59); *P* < 0.0001]. The analgesia consumption was significantly reduced in the DEX group compared with the MID group [mean (SD): 5.37 (4.64) vs 10.09 (7.24); *P* < 0.0001). There was no significant difference in terms of functional recovery (*P* = 0.180) and length of hospital stay (*P* = 0.385) between the two groups (Table 2).

**Table 2:** Postoperative outcome of the DEX versus MID groups

Outcome Variable ^a^	DEX group (*n* = 88)	MID group (*n* = 88)	t	*P*-value
VAS_MAX_ Score ^b^	0.28 (0.86)	2.26 (2.59)	4.816	0.0001
Accumulation of analgesia consumption, number ^c^	5.37 (4.64)	10.09 (7.24)	2.428	0.0001
Functional recovery, hours ^d^	20.76 (4.01)	21.51 (4.86)	1.465	0.180
Hospital stay, day	7.34 (2.50)	7.89 (3.70)	2.202	0.385

VAS_MAX_ Score: maximum postoperative visual analogue scale score of each patient

^a^ Data are expressed as mean (SD)

^b^ Indicates the maximum visual analogue scale (VAS) score of patients in hospital

^c^ Accumulation of analgesia consumption was used to describe the accumulation of the analgesia (oral administration of oxycodone 5 mg once daily) postoperatively

^d^ Functional recovery means that patients can walk with functional aids and complete functional exercise

## Discussion

When K–M analysis was performed on patients systemically administering DEX during PNB, the DEX group had a higher ratio of patients who attained the target IOC2 (i.e. mean high analgesic efficacy) in a significantly faster manner than the MID group.

To the best of our knowledge, this is the first study to assess and confirm the time required to attain the IOC2 target with DEX administration. In our study, we found that this time was approximately 15 min. In a previous pharmacokinetics report, non-compartmental analysis revealed that the distribution half-life of DEX was approximately 6 min [18], and its elimination half-life was 2.1–3.1 h [19]. In line with the findings of our study, DEX has been shown to have a great intrinsic analgesic effect [20, 21]. In contrast, MID has no analgesic effect, has decreases the pain threshold and increases pain perception [22]. Several theories have been proposed to elucidate these analgesic properties of DEX. The significant anti-nociceptive effects of the systemic administration of DEX are mainly attributed to the reduction of sympathetic tone [23]. DEX acts through both pre- and post-synaptic sympathetic nerve terminals; it decreases the sympathetic outflow and norepinephrine release, leading to its anxiolytic and analgesic effects [24]. The strong inflammatory responses that affect the development of pain can be controlled effectively by DEX [25]. DEX has analgesia-sparing effects that act centrally in the locus ceruleus [26] and a unique analgesic effect that acts on neuropathic pain with emotional components [27]. Further, DEX pharmacologically binds to pre-synaptic C-fibres, and the potential targets of DEX are post-synaptic neurons in the posterior horn of the spinal cord. Thus, it effectively antagonises the activation of peripheral nociceptors [28]. Lastly, neuraxial DEX can also inhibit the activation of spinal microglia and astrocytes as well as reduce the release of noxious substances caused by noxious stimulation. Moreover, it blocks the crosstalk between spinal neurons and glia cells under chronic pain conditions and regulates the transmission of noxious information [29, 30]

An ideal analgesic agent should provide satisfactory pain relief without side effects in patients. For respiratory safety, patients treated with DEX were less likely to experience hypoxemia, owing to the potential respiratory system stress effect of DEX, the SpO_2_level briefly decreased to 93%; and it rapidly recovered to > 95% in most patients in our study. In contrast, MID, as one of the classic sedatives, is well known for causing respiratory depression and even has the potential to cause apnoea [31]. In our trial, more than half of the patients treated with a relatively high dosage of MID experienced hypoxemia. None of the clinically significant side effects, such as hypotension, hypertension and bradycardia, associated with DEX were observed in our trial. Due to employ continuous infusion at a constant speed without loading dosage infusion to avoid reaching high peaks of plasma concentration, which resulted in a stable and adequate plasma level uptake. Additionally, the median attainment time of the target sedation was 17 min in the DEX group in our study, which is in line with the results of a previous study claiming that DEX can rapidly attain the sedation level and effect-site concentration peak in approximately 13 min [32, 33]. In our trial, patients in the DEX group did not exhibit notable agitation, which may be because DEX provides greater muscle relaxation than MID, as observed in the EMG results. Some trials in line with our result and have reported that MID can cause agitation and restlessness, which can affect the ongoing procedure [34, 35]. DEX can be used to reduce postoperative pain, which is also important for patient satisfaction. In our trial, intraoperative DEX effectively reduced the pain VAS score, which in turn reduced postoperative analgesia consumption during the recovery period of the patients. The parameters such as sex, age, ASA score and BMI did not affect the IOC2 target attainment after DEX administration.This suggests that even in subgroup populations, there is no great difference in using DEX for analgesia and achieving ideal analgesic effect.

Recently, several methods of pain monitoring have been developed [36]. In our study, analgesia nociception index and surgical pleth index based on peripheral (sympathetically mediated) vasoconstriction and cardiac autonomic tone were easily affected by vasoactive drugs, particularly in patients with trauma. The most common opioids have the greatest impact on pupil monitoring (pupillometric assessment of nociception). The reaction of skin sweat glands is affected by high interference [36]. NFR threshold is affected by the femoris muscle, which is further affected by PNB. None of the above parameters were suitable for detecting the level of analgesia in our study. Based on the understanding of nociception relative to the nuclei of the brain in a recent study, we selected IOC2 (qNOX), a EEG-based score, because it does not rely on a measure of (peripheral) autonomic activity and is more robust against the influence of cardiovascular medications and co-morbidities. (http://quantiummedical.com/products/qcon2000/). Jensen et al. and Melia et al. have reported that IOC2 derived from EEG signals has also been proposed as a non-invasive guide to indicate the depth of anaesthesia [11, 12]. Our previous study found that predictive qNOX detects hypothermia and has a potentiating effect on the depth of analgesia [9]. IOC2 detected propofol and sevoflurane provided better analgesia, an effective method to reduce stress and the intraoperative nociceptive stimulus response [10]. However, another study [37] showed a lack of correlation between the IOC2 (qNOX) value and postoperative pain; they included different types of surgery with significantly different levels of noxious stimulation. This led to various confounding factors for IOC2 for more specific types of pain. To avoid the same issue, our study unified the type and location of surgery. Further, we used the exact dosage of DEX and MID in our study. IOC2 is a variable strongly influenced by the depth of anaesthesia [11, 12, 38]. As recommended by the manufacturer, it is necessary to ensure the reliability of IOC2 under the condition of sedation. We reported IOC1 values simultaneously to ensure the accurate assessment of IOC2 as quality control.

This study has some limitations. First, we did not evaluate the long-term outcomes of DEX administration, including chronic pain after discharge. Large-scale studies are required to rule out any long-term adverse effects after discharge. Second, the population included relatively healthy and young patients; thus, the effect of DEX administration in children and medically compromised populations is yet to be investigated. Future trials should investigate the effect of DEX administration in these populations.

## Conclusions

This trial using the IOC2 could evaluate the independent analgesic efficacy of DEX in real-time. Our results support the systemic administration of DEX as a useful adjuvant of PNB to alleviate intraoperative pain and discomfort. DEX was determined to provides fast and appropriate intraoperative analgesia and effective postoperative analgesia along with significant haemodynamic stability, mild decrease in HR and effective muscle relaxation without respiratory depression.

## Data Availability

The raw data of this study are available from the corresponding author on. reasonable request.
